# Supplementary material for: Evaluating the Effectiveness of Water-Saving Irrigation on Wheat (Triticum aestivum L.) Production in China: A Meta-Analytical Approach
Source: Plants (Basel). 2025 Sep 11;14(18):2837. doi: 10.3390/plants14182837 (PMC12473444; doi:10.3390/plants14182837)
Supplement: Supplementary file 1 [file plants-14-02837-s001.zip › plants-supplementary materials.pdf]

Table S1. Egger's regression tests and fail-safe numbers for assesssing publication bias#

Var.	k	Rosenthal's method
Overall-yield	422	112593936
Drip irrigation-yield	308	36006768
Micro-sprinkler irrigation-yield	102	21129793
Overall-WUE	305	501858
Drip irrigation-WUE	218	206591
Micro-sprinkler irrigation-WUE	79	25256
Overall-PFPN	367	31117
Drip irrigation-PFPN	261	16458
Micro-sprinkler irrigation-PFPN	94	1345

No publication bias if the conservative cutoff value for Rosenthal's method is > 5k+10.

Table S2. Heterogeneity test statistics for pooled and subgroup meta-analyses (Q_t and *p*-values)

Var.	Q_t	*p*-values
Overall-yield	89747546.6749	<0.0001
Drip irrigation-yield	9426180.1691	<0.0001
Micro-sprinkler irrigation-yield	13645841.9357	<0.0001
Overall-WUE	9075.4165	<0.0001
Drip irrigation-WUE	5358.4921	<0.0001
Micro-sprinkler irrigation-WUE	2377.1379	<0.0001
Overall-PFPN	1538.1317	<0.0001
Drip irrigation-PFPN	1400.7808	<0.0001
Micro-sprinkler irrigation-PFPN	697.49	<0.0001

Q_t = Cochran's total Q for heterogeneity; Bold p<0.05.

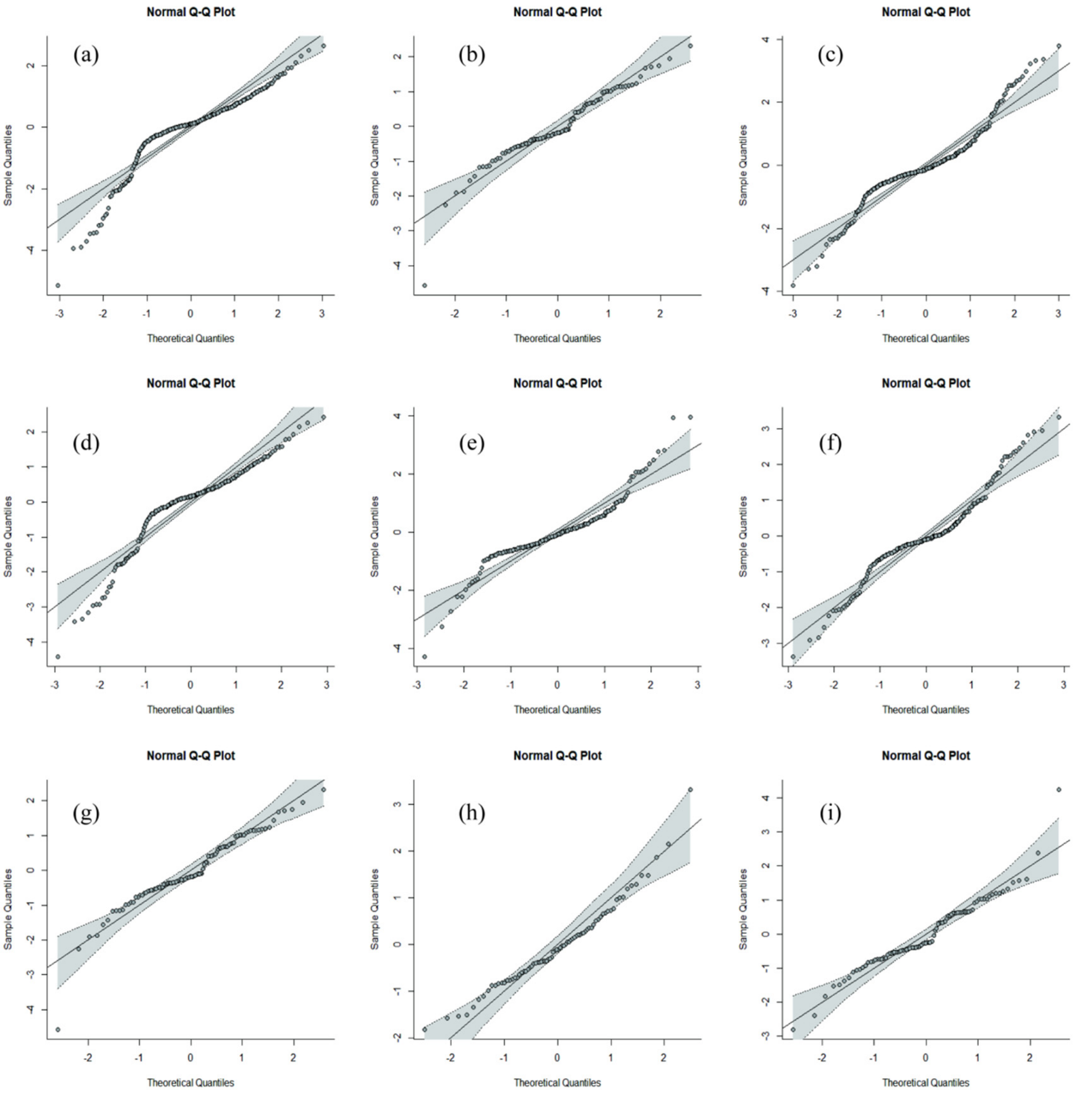

Figure S1. Normal quantile–quantile (Q–Q) plots for assessing publication bias. Panels (a–c) show results for all data combined, (d–f) for drip irrigation, and (g–i) for micro-sprinkler irrigation.
